# Contribution to a Sustainable Society: Biosorption of Precious Metals Using the Microalga *Galdieria*

**DOI:** 10.3390/ijms25020704

**Published:** 2024-01-05

**Authors:** Eri Adams, Kazuki Maeda, Yuki Kamemoto, Kazuho Hirai, Egi Tritya Apdila

**Affiliations:** Galdieria, Co., Ltd., Yokohama 2300046, Kanagawa, Japanegi_apdila@galdieria.com (E.T.A.)

**Keywords:** *Galdieria*, gold, palladium, urban mining, ASGM, double cropping, ergothioneine

## Abstract

The red microalga *Galdieria* sp. is an extremophile that inhabits acidic hot sulphur springs and grows heterotrophically to a high cell density. These characteristics make *Galdieria* suitable for commercial applications as stable mass production is the key to success in the algae business. *Galdieria* has great potential as a precious metal adsorbent to provide a sustainable, efficient and environmentally benign method for urban mining and artisanal small-scale gold mining. The efficiency and selectivity in capturing precious metals, gold and palladium from metal solutions by a *Galdieria*-derived adsorbent was assessed relative to commercially used adsorbents, ion exchange resin and activated charcoal. As it is only the surface of *Galdieria* cells that affect metal adsorption, the cell content was analysed to determine the manner of utilisation of those metabolites. *Galdieria* was shown to be protein-rich and contain beneficial metabolites, the levels of which could shift depending on the growth conditions. Separating the cell content from the adsorbent could improve the adsorption efficiency and reduce CO_2_ emissions during the metal collection process. The commercial applications of *Galdieria* appear promising: growth is quick and dense; the precious metal adsorption capacity is highly efficient and selective in acidic conditions, especially at low metal concentrations; and the cell content is nutrient-rich.

## 1. Introduction

Biosorption is a technique to collect a substance of interest using organism-derived matter, either dead or alive [1]. Biosorption is considered to be an eco-friendly and often cost-effective alternative to current wastewater treatment techniques. Despite the increasing attention this process has attracted due to pressing environmental concerns, very few large-scale industrial applications are in operation. This could be attributed to technical challenges such as the availability of the biomass in a timely manner and physical restrictions in upscaling laboratory-scale cultivation systems to a commercial capacity. Other examples of biological strategies against environmental concerns involve biodegradation and phytoremediation [2,3].

The geographic locations where precious metals such as gold (Au) and palladium (Pd) can be mined are limited. To satisfy the increasing demand and potential risks to supply chains due to unpredictable global political and economic changes, precious metal recovery through recycling is becoming ever more important. Waste containing precious metals such as electrical and electronic waste (e-waste) is termed “an urban mine” and this often contains higher concentrations of metals than natural mines. Currently, only 15–20% of e-waste is recycled; the rest goes into landfills as recovering precious metals from diverse composites of all sorts of materials to the high levels of purity required in the industry in a safe and cost-effective manner is highly challenging [4]. It is also possible that the residual precious metals in landfills cause toxicity to the ecosystem [5]. Biosorption has the potential to replace current metal adsorbents such as ion exchange resin and activated charcoal with more sustainable and efficient materials and increase precious metal recycling ratios. A biosorbent derived from *Galdieria*, a red (Rhodophyta) microalga, has been shown to possess a superior ability to recover precious metals such as Au and Pd [6]. *Galdieria* is an extremophile that thrives in hot sulphur springs, tolerating acidity as low as pH 0.2 and moderately high temperatures up to 57 °C [7]. *Galdieria* is also capable of growing heterotrophically to a high cell density as the organism can feed on an exogenous carbon source instead of performing photosynthesis to generate its own carbon source. Carbon sources on which *Galdieria* can live span over 50 different types [8,9,10,11]. These characteristics make *Galdieria* a promising candidate for mass production. Our previous work demonstrated the mechanism of precious metal adsorption onto the surface of *Galdieria* cells [12]. In short, X-ray absorption fine structure (XAFS) measurements indicated that the primary site of Au and Pd adsorption was the nitrogen atom on the cell surface in a chlorine (Cl)-rich environment where enough Cl atoms were available for the precious metals to form chloride anionic complexes. Although Pd remained ionic (divalent), Au underwent progressive bio-reduction to metallic Au(0) by switching binding partners, possibly promoted by sulphur oxidation, the mechanism that contributes to adsorption efficiency. The high specificity of the *Galdieria* adsorbent to precious metals among the other base metals and the high tolerance to strong acidity is especially beneficial in the urban mining sector where industrial wastewater is often highly acidic with high concentrations of base metals and low concentrations of precious metals. Here, we demonstrate the ability of a *Galdieria* adsorbent and the advantages of the biosorbent in terms of an industrial application.

*Galdieria* is also known to be rich in protein and polysaccharides [13] and contains various beneficial metabolites such as a blue pigment, phycocyanin, as well as an antioxidant, glutathione [14,15]. As the cell surface alone is sufficient for precious metal adsorption, a cell content analysis of the strain NS3 was performed to determine the manner of utilisation. It was found that the composition of the cell content changed, depending on the mode of cultivation. The cells were also shown to contain high-value beneficial compounds such as ergothioneine and highly branched glycogen. By utilising the cell content, an additional contribution to environmental sustainability is expected over and above improved metal adsorption efficiency and reduced carbon emissions at the end of the metal recovery process, let alone the potential health and medical benefits of the metabolites themselves.

*Galdieria* sp. NS3 was demonstrated to be a promising candidate for commercial applications because (1) rapid mass production with low biological contamination risks is possible at a 1000 L scale; (2) the precious metal adsorption ability, especially in high acidities (as high as 6 M aqua regia), low precious metal concentrations (especially below 10 mg/L) and base metal mixtures, is equivalent or superior to current methods; and (3) the cells contain various valuable metabolites, including protein and carbohydrate, the composition of which could be altered to a more favourable direction by modifying the mode of cultivation.

## 2. Results

### 2.1. Large-Scale Production of Galdieria

**Figure 1:**
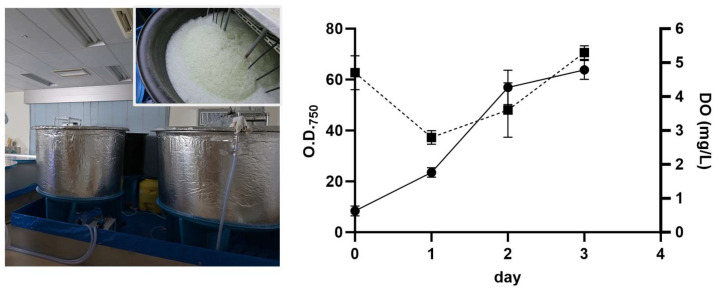
*Galdieria* cultivation facility at Galdieria, Co., Ltd., Yokohama, Japan, and its growth pattern. Optical density (O.D._750_; solid line) and dissolved oxygen (DO) values (dashed line) are shown. Error bars indicate standard error (*n* = 5).

Indoor cultivation facilities with 1000 L cultivation tanks were established and the production of more than 200 kg dry weight per month of *Galdieria* sp. strain NS3 was achieved using a semicontinuous cultivation system (Figure 1). The mode of cultivation was heterotrophic, with commercial sugar as the carbon source. The growth media used was a modified Allen medium [12]. Growth peaks after 2 to 3 days were measured using optical density at 750 nm (O.D._750_) accompanied by dissolved oxygen (DO) values, which decreased during active growth due to the respiration of *Galdieria* and then resumed to the basal level once growth slowed. Reusing the cultivation medium saved freshwater use and the costs involved in wastewater disposal (such as neutraliser). After harvesting the cells through continuous centrifugation, the slurry underwent adsorbent production processes involving desiccation and powderisation. As it is the natural cell surface property of *Galdieria* that facilitates the efficient adsorption of precious metals, extensive pre-processing and costly modifications were not required.

### 2.2. Precious Metal Collection Capacity of Galdieria in Urban Mines

**Figure 2:**
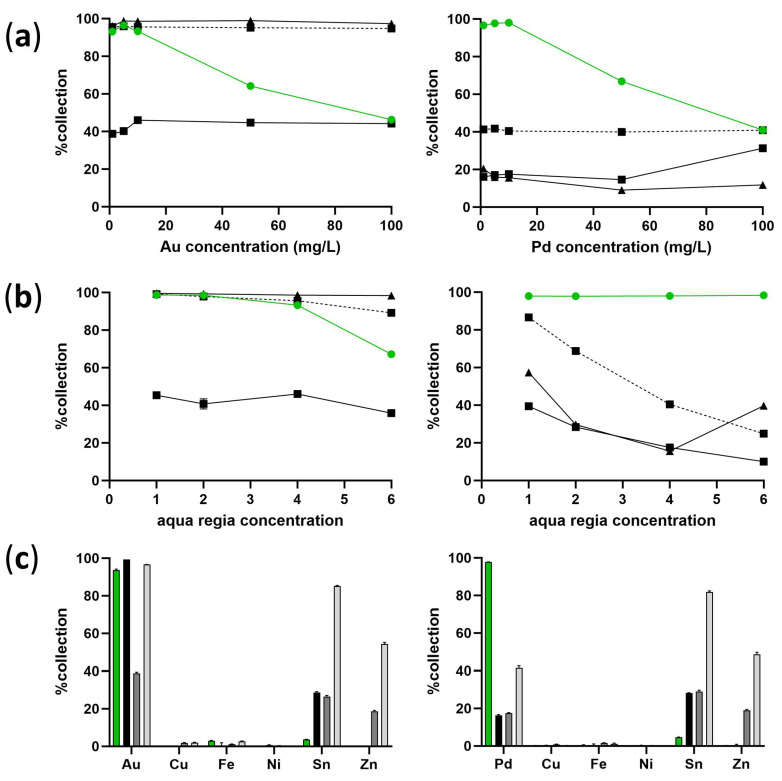
Metal adsorption efficiency of *Galdieria* relative to currently used adsorbents. (**a**) %Collection of Au and Pd from 4 M aqua regia solution containing 1–100 mg/L metal. (**b**) %Collection of Au and Pd from 1–6 M aqua regia solutions containing 10 mg/L metal. (**c**) %Collection of Au and Pd from 4 M aqua regia solution containing 10 mg/L each of indicated metals. 10 g/L for *Galdieria* (green line or bar), activated charcoal (triangle or black bar) and ion exchange resin (square or dark grey bar) were homogeneously mixed in a metal solution for 5 min with vigorous shaking and separated from the solution prior to metal quantification. Ion exchange resin (10 g/L) was also incubated for 1 h in the metal solution with vigorous shaking prior to quantification (dashed line or light grey bar). Error bars indicate standard error (*n* = 3).

Using the biosorbent produced as described in the previous section, its capacity as a precious metal adsorbent was assessed and compared with ion exchange resin and activated charcoal methods. As shown in Figure 2a, a *Galdieria*-derived adsorbent demonstrated a near-complete recovery of Au or Pd at concentrations below 10 mg/L after the homogeneous mixing of the adsorbent and the metal solution. Under these conditions, ion exchange resin, the most commonly used adsorbent in the current system, was outperformed by *Galdieria*. Increasing the time from initial mixing to 1 h dramatically improved the performance of ion exchange resin for Au recovery to equivalent to the level of activated charcoal, but not significantly for Pd. Aqua regia is an extremely strong acid often used to dissolve scrap metal to facilitate precious metal extraction, and the *Galdieria*-derived adsorbent was shown to function well in concentrations as high as 6 M aqua regia, especially for Pd recovery (Figure 2b). Improved Pd adsorption efficiency for activated charcoal at 6 M aqua regia may have been due to the increased pore volume and functional groups on the surface [16,17]. The recovery ratios of *Galdieria* were equivalent to those of activated charcoal or ion exchange resin incubated for 1 h for Au and up to 4 M aqua regia, much better than any other adsorbents tested for Pd. The *Galdieria*-derived adsorbent was also tested in a solution containing base metals (Figure 2c). Although base metal adsorption was kept low for *Galdieria*, activated charcoal adsorbed approximately 30% tin (Sn) and ion exchange resin adsorbed approximately 20% zinc (Zn) in addition to Sn. Ion exchange resin incubated for 1 h showed an even higher adsorption of Sn (approximately 85%) and Zn (approximately 55%).

**Figure 3:**
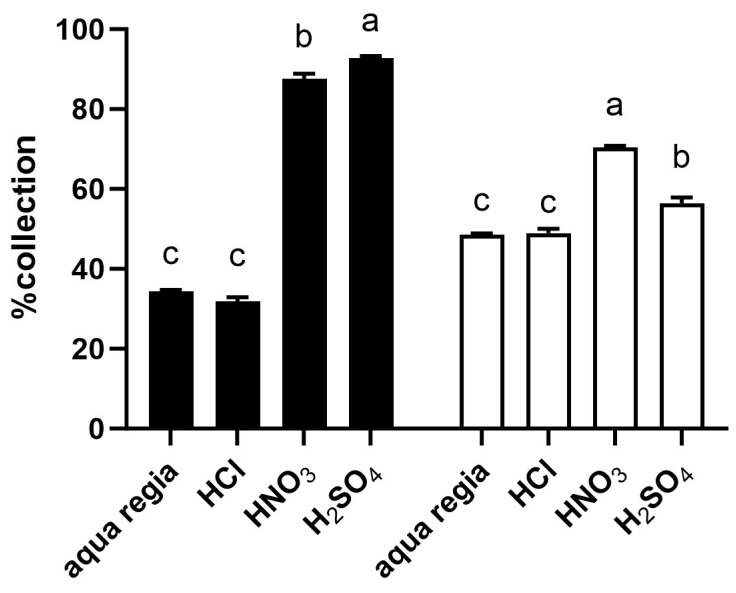
Adsorption efficiency at low metal concentrations in various acids. %Collection of Au (black) and Pd (white) from 1 M acid solution containing 10 mg/L metal. *Galdieria* (1 g/L) was incubated for 5 min in a metal solution with vigorous shaking. Error bars indicate standard error (*n* = 3) and letters indicate statistical differences (*p* < 0.05).

To further assess the capacity of *Galdieria* in urban mining, the *Galdieria*-derived adsorbent was tested in various acid solutions containing a low concentration of metal (10 mg/L). Although the near-complete recovery of Au or Pd was achieved with 10 g/L *Galdieria*, as shown in Figure 2, reducing the amount of the *Galdieria* adsorbent to 1 g/L highlighted the differences among the acids, especially for Au recovery (Figure 3). The recovery ratios were much higher for the acids that promote bio-reduction (such as sulphuric acid (H_2_SO_4_) and nitric acid (HNO_3_)) compared with aqua regia or hydrochloric acid (HCl) during Au recovery. In contrast, a slightly better recovery for Pd was observed in HNO_3_ and H_2_SO_4_, though the difference was relatively small.

### 2.3. Ability of Gold Recovery in ASGM

**Figure 4:**
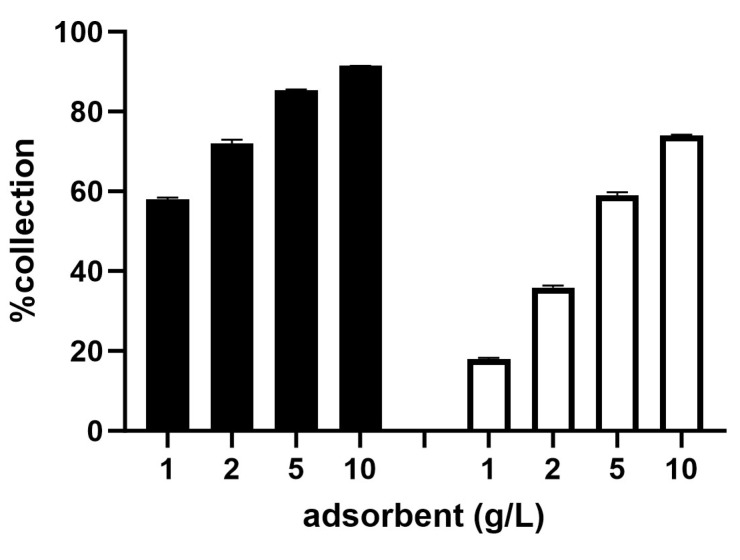
System validation of the iodine–*Galdieria* method. %Collection of Au from a mock solution with 2.97 mg/L Au powder using 1–10 g/L of dried *Galdieria*-derived adsorbent (black bars) or equivalent amount of *Galdieria* slurry (white bars). Error bars indicate standard error (*n* = 3).

Our *Galdieria*-derived adsorbent technology for precious metal recovery is not only applicable for use in urban mining but is also applicable to commercial ore mining operations. Artisanal and small-scale gold mining (ASGM) accounts for nearly 40% of anthropogenic mercury emissions in the world [18]. In order to reduce the health risks and environmental damage associated with the use of mercury in Au recovery, we developed a method to extract Au from ore powder using an iodine solution to facilitate the adsorption by *Galdieria*. Figure 4 shows the results of a mock experiment using dissolved Au powder and *Galdieria* as an adsorbent. Au powder was dissolved in an aqueous iodine solution to obtain a 2.97 mg/L Au solution and the solution was reduced using L-ascorbic acid. Au was collected by adding either 1–10 g/L of the dried *Galdieria*-derived adsorbent or an equivalent amount of *Galdieria* slurry to the reduced solution. The slurry was prepared by separating fully grown *Galdieria* from the growth medium through centrifugation. Au was successfully recovered from the iodine solution by the *Galdieria* adsorbent in a dose-dependent manner, thus validating the technology. Although the experiment using slurry showed a somewhat lower efficiency than the dried and powdered adsorbent, the *Galdieria* slurry was indicated as possessing a sufficient adsorption ability for Au.

**Figure 5:**
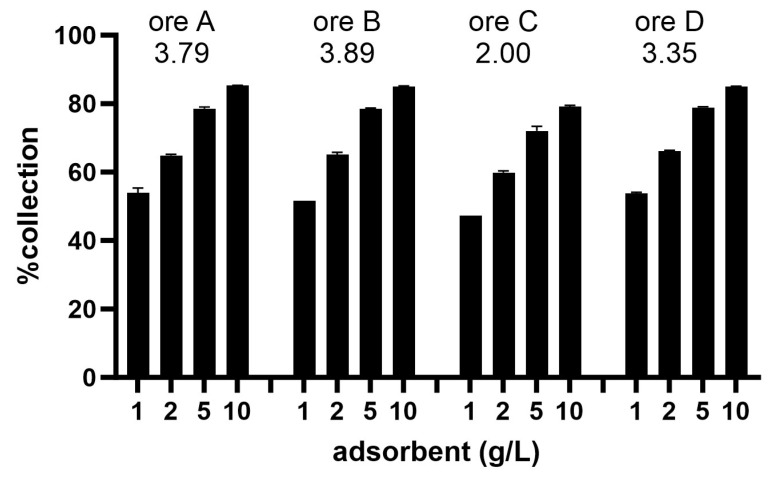
Au collection from ores in Kenya. %Collection of Au from ore extract in iodine solution using 1–10 g/L of the *Galdieria*-derived adsorbent. Numbers indicated below each ore are the Au contents in g Au/t ore. Error bars indicate standard error (*n* = 3).

As the iodine–*Galdieria* method was demonstrated to be effective, the method was tested using real Au ore powder from mining sites in Kenya (Figure 5). An iodine solution was added to finely ground ore and incubated overnight on a rotator. The iodine solution successfully extracted Au from four different types of ores, ranging from 2.00 to 3.89 g Au/t ore. Next, 1–10 g/L of the *Galdieria*-derived adsorbent was added to the reduced Au solution and good adsorption efficiencies were observed for all tested ores in a dose-dependent manner. The efficiencies were equivalent to those obtained in a mock solution (Figure 5), suggesting the practical suitability of the technology.

### 2.4. Content Analysis of Galdieria Cells Grown in Heterotrophy, Mixotrophy and Autotrophy

**Figure 6:**
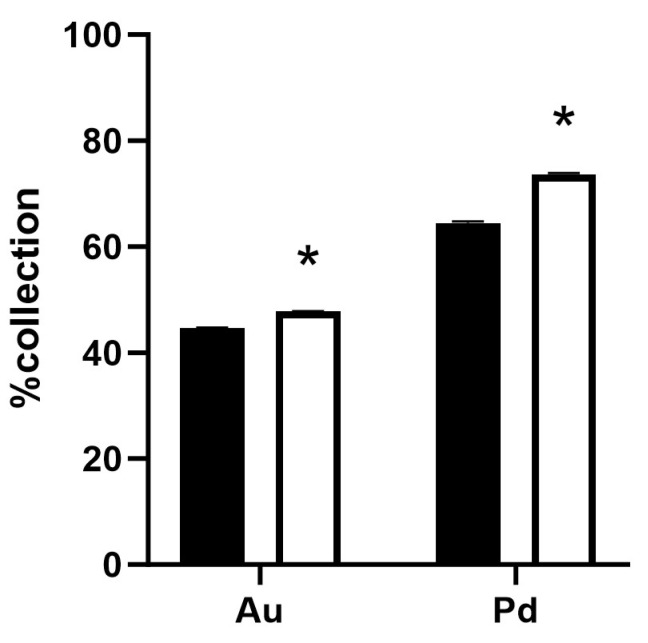
Effects of cell content extraction from *Galdieria*. %Collection of Au and Pd from 4 M aqua regia solution containing 100 mg/L of each metal using 20 g/L post-extraction adsorbent (white bars) or native control (black bars). Error bars indicate standard error for technical replicates (*n* = 3) and asterisks indicate statistical significances (*p* < 0.01).

A method to reduce the carbon footprint from the metal recovery process involving the *Galdieria*-derived adsorbent could be by extracting carbon-rich metabolites that do not participate in metal adsorption prior to the preparation of the adsorbent. Microalgae are known to contain a range of bioactive molecules that are potentially useful or beneficial for humans such as macronutrients, vitamins, minerals, antioxidants and pigments [19]. The extraction of metabolites reduces the adsorbent mass as well as CO_2_ emissions during combustion. The grinding of dried *Galdieria* and its subsequent extraction with water yielded a *Galdieria* extract and residual slurry. The slurry was dried, ground and tested for its metal adsorption capacity. The post-extraction *Galdieria* was found to increase adsorption efficiency per weight relative to the non-extracted adsorbent (Figure 6).

Next, we analysed the cell contents of *Galdieria* grown in heterotrophic, mixotrophic and autotrophic modes of nutrition. As shown in Table 1, increased levels of protein and fat were detected in the order of autotrophy, heterotrophy and mixotrophy. In reverse, decreased levels of carbohydrate were found in the same order, of which the sugar level was below the limit of detection in autotrophically grown *Galdieria*.

**Figure 7:**
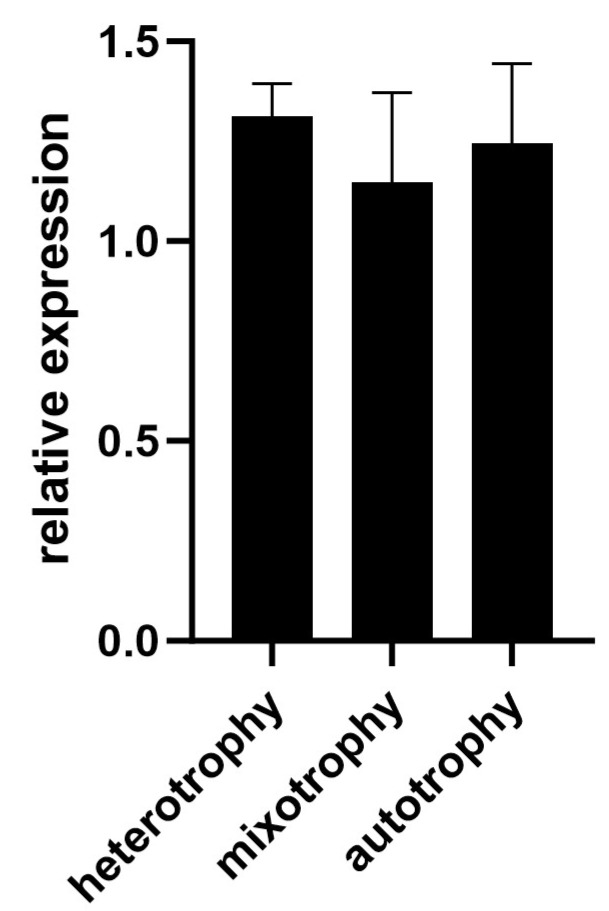
*Argininosuccinate synthase* expression in *Galdieria* grown in heterotrophic, mixotrophic and autotrophic modes. Error bars indicate standard error (*n* = 3).

Further analysis on the hydrolysed amino acid profile revealed the highest values for all the amino acids tested from autotrophically grown cells (Table 2), consistent with the results from Table 1. An increase of more than 2.5 times was observed for autotrophically relative to heterotrophically grown *Galdieria* for Asp + Asn, Ala, Arg, Ile, Met and Leu. Of these, Ile, Met and Leu are essential amino acids. Slightly higher levels of amino acids were attained in mixotrophically grown *Galdieria* compared with heterotrophically grown *Galdieria*. Argininosuccinate synthase is the rate-determining enzyme in an Arg biosynthetic pathway. The gene expression of a putative gene encoding argininosuccinate synthase (Gasu_30780) in *Galdieria* sp. NS3 grown in heterotrophic, mixotrophic and autotrophic modes was analysed; however, the expression levels were found to be high and constant among the conditions tested (Figure 7).

Next, ergothioneine, a non-proteinogenic amino acid that possesses strong antioxidising properties, was quantified in *Galdieria* cells (Table 3). Interestingly, approximately 35–39 mg/100 g ergothioneine was detected in *Galdieria*, irrespective of the growth mode.

Of the various candidate metabolites in *Galdieria* with a market potential, our preliminary in-house trial for the functional analysis of a polysaccharide, glycogen, indicated an improved intestinal bacteria diversity index and increased so-called “beneficial bacteria” such as *Bifidobacterium*, *Coprococcus* and *Blautia*.

## 3. Discussion

As an extremophilic red microalga, *Galdieria* can grow under an autotrophic mode of nutrition through photosynthesis, the mode that is mainly undertaken in its natural habitat, as well as under a heterotrophic mode of nutrition where a carbon source is provided in the absence of light. *Galdieria* is known to utilise a variety of carbon sources and is able to grow to high cell densities in heterotrophic conditions relative to other microalgae [20,21]. As *Galdieria* is highly tolerant of strong acidities and moderately high temperatures, it can outcompete other organisms by habitat segregation. *Galdieria* is also able to survive in high salt concentrations equivalent to seawater [22]. The use of seawater to cultivate this alga could reduce the cost of using freshwater and further reduce the risk of biological contamination as there are no natural habitats on the earth landmass that host seawater as acidic as *Galdieria*’s habitat. These characteristics of *Galdieria* are convenient and make *Galdieria* suitable for industrial large-scale open cultivation without the risk of biological contamination, which is the key to success in the algae industry.

*Galdieria* sp. NS3 was shown to be suitable for mass production, demonstrating fast growth to a high cell density. Using our method in 1000 L cultivation tanks, we achieved a final O.D._750_ of around 60–70, which is equivalent to approximately 20 g/L dry weight of cells, within 60 h (Figure 1). This is the first report of high-density *Galdieria* cultivation at this scale as far as our knowledge extends. However, at a much smaller scale (1.5 L culture volume), the achievement of 80–120 g/L dry weight was reported for another strain (074G) in a fed-batch culture [23], suggesting room for improvement.

The *Galdieria*-derived adsorbent was demonstrated to be particularly suitable for adsorption in acidic solutions with low precious metal concentrations (<10 mg/L), which, currently, are often discarded (Figure 2). *Galdieria* is highly tolerant to acidity, probably because it naturally inhabits acidic springs. As strong acids, including aqua regia, are often used to dissolve scrap metal, wastewater is commonly acidic. The acid-tolerant nature of *Galdieria* is a great advantage in the metal recovery process. Another advantage of the *Galdieria*-derived adsorbent is that the metal binding reaction is fast. The near-complete recovery of Au or Pd at concentrations below 10 mg/L was readily achieved after homogeneously mixing the adsorbent and the metal solution. In contrast, ion exchange resin only recovered approximately 40% or 20% of Au or Pd shortly after mixing, respectively. Incubation for 1 h increased the efficiency to an equivalent level as for the *Galdieria*-derived adsorbent for Au but only to 40% recovery for Pd. It is assumed that the mechanical distribution of nitrogen atoms, the initial binding sites of Au and Pd, on the *Galdieria* cell surface is particularly suitable for complex adsorption [12]. A high specificity to precious metals among base metals is also an advantage of the *Galdieria*-derived adsorbent due to its surface state, which attracts negatively charged precious metal complexes rather than positively charged base metals. Although activated charcoal or ion exchange resin incubated for 1 h seemed suitable for the recovery of Au in acidic wastewater at low precious metal concentrations, selectivity towards Au among base metals was compromised relative to *Galdieria*. As urban mines typically contain much higher concentrations of base metals relative to precious metals, which are the targets for recycling, the high specificity of the *Galdieria*-derived adsorbent is highly favourable.

Analyses with various acids revealed that for Au recovery, a better performance was observed in a reducing environment such as H_2_SO_4_ and HNO_3_ compared with HCl and aqua regia at 10 mg/L Au or Pd (Figure 3). This tendency was consistent with the observation from our previous findings at much higher metal concentrations (1 g/L). For Au, the progressive reduction of ionic Au to metallic Au vacated the binding positions on the cell surface to upcoming ionic Au, resulting in improved adsorption efficiency. Meanwhile, Pd remained in an ionic state after adsorption; therefore, the adsorption efficiency did not depend much on the nature of the solvent [12]. This knowledge may assist with the choice of acid for efficient metal recovery in urban mining.

The *Galdieria*-derived adsorbent is not only suitable for metal recovery through recycling but is also promising for metal recovery from natural ores. Au mining is known to utilise deleterious chemicals such as mercury and cyanide to refine Au. Of these, mercury is a particularly dangerous neurotoxin that can harm humans and wildlife. It is exemplified in Minamata disease, and can be widely distributed via the air, water and land once emitted, thus contaminating the ecosystem. Our aim is to offer an alternative environmentally benign method utilising *Galdieria* for these deleterious chemicals, especially mercury. We developed a two-step method: (1) eluting Au from ore using an iodine solution; and (2) collecting Au from the reduced eluate using *Galdieria*. This new methodology proved to possess good Au recovery efficiency (80–90% recovery with 10 g/L adsorbent), both in a mock Au solution and in real ore eluates (Figure 4 and Figure 5). The efficiency among four different ores tested was equivalent, suggesting the applicability of the iodine–*Galdieria* method for various types of ores. The use of *Galdieria* slurry immediately after the removal of the growth medium compromised Au recovery by approximately 17% relative to the dried and powdered *Galdieria* adsorbent at 10 g/L. However, this finding might be particularly important at ASGM sites where the processing of adsorbents through desiccation and grinding is not easily achieved or would be prohibitively expensive. Although the iodine–*Galdieria* system technically worked very well, a downside could be the high price of iodine, which would be a barrier to its adoption at ASGM sites. We are currently working on reducing the cost of extraction solutions by rejuvenating the iodine eluate to a form available for additional Au extraction.

In nature, *Galdieria* endolithically grows in hot sulphur springs under an autotrophic mode, performing photosynthesis by absorbing sunlight and CO_2_. As is the case for most plant species, approximately half of the cell weight in *Galdieria* is carbon. At the end of the metal recovery process, *Galdieria* bound to precious metals is combusted to obtain pure metals, during which half of the adsorbent is converted to CO_2_ and emitted into the atmosphere. If we could shift the cultivation mode from a heterotrophic (where carbon sources (not CO_2_) are utilised) to an autotrophic mode (where CO_2_ is consumed), the CO_2_ emitted from the combustion of *Galdieria* could be cancelled out by the CO_2_ absorbed through cultivation. However, the downside of autotrophic cultivation is the slow pace of growth and the difficulties in scaling-up production as the larger the system and the denser the culture, the greater the challenge of accessibility to CO_2_ and light for each cell. Technology to improve the autotrophic growth rate by modifying the way CO_2_ is made available and to introduce light into the growth medium is awaited.

Alternatively, the carbon-rich cell content that does not participate in metal adsorption can be excluded from the adsorbent as the precious metal adsorption process solely occurs on the surface of the cell [12]. The extraction of the cell content improved the metal adsorption efficiency per weight of adsorbent as expected (Figure 6). By commercialising the cell contents, the cost of producing the adsorbent could be reduced. Promoting metal recycling is the next major challenge.

A content analysis of *Galdieria* sp. NS3 revealed major metabolic shifts, depending on the growth mode. In autotrophy, high protein (64.2%) and fat (8.3%) contents and a low carbohydrate (22.8%) content were recorded, while the reverse pattern was observed in heterotrophy (low protein (37.9%) and fat (4.9%) contents and a high carbohydrate (50.6%) content) (Table 1). Mixotrophically grown cells showed similar characteristics to heterotrophic cells. This tendency has been shown in a previous publication on another ACUF strain of *Galdieria*, with 26.5% protein, 1.1% lipid and 69.1% carbohydrate in heterotrophy and 32.5% protein, 1.8% lipid and 62.9% carbohydrate in autotrophy [13]; however, the NS3 strain was shown to contain more protein in general and had a tendency to shift metabolic pathways more drastically among the different modes of nutrition. Our content profile in autotrophy was closer to another strain (074G) grown heterotrophically with a high cell density; this was then illuminated with a high light to accumulate phycocyanin [24]. Soybean, which is generally considered to be protein-rich, has been reported to contain 35.3–37.2% protein in grains [25], a level equivalent to or lower than the heterotrophically grown *Galdieria*. Consistent with the protein contents obtained in Table 1, the amino acid profile also showed a tendency towards high overall amino acid contents in autotrophy relative to heterotrophy (Table 2). This was not the case in the previous reports on strains ACUF 064 and SAG 108.79, where they showed equivalent amino acid profiles between autotrophic and mixotrophic *Galdieria* [26,27]. Abundance ratios for amino acids are more or less similar among the *Galdieria* strains reported, except extremely low Cys was detected in the strain UTEX 2919 [28]. A nitrogen-rich amino acid, Arg, was found to be 2.73 times higher in autotrophically grown NS3 compared with heterotrophically grown cells. Although the gene encoding argininosuccinate synthase, the rate-determining step of Arg biosynthesis, is known to be regulated at the transcriptional level in Arabidopsis [29], its expression in *Galdieria* was constant among the three different modes of growth (Figure 7), indicating that this was not the regulative point for a high accumulation of Arg in autotrophy. Further research on the mechanism of how autotrophically grown *Galdieria* accumulates high levels of protein/amino acids is much anticipated.

Ergothioneine is a rare amino acid that occurs only in certain species such as cyanobacteria and fungi. It has recently gained popularity in the food industry as it can only be acquired through diet in humans. The emerging outcomes point towards health-promoting benefits, mainly due to its powerful antioxidant effects [30]. Interestingly, ergothioneine was detected in *Galdieria* sp. NS3 grown in all trophic modes tested (Table 3). The levels did not change depending on the modes of nutrition, and 35–39 mg ergothioneine/100 g *Galdieria* dry mass is a level that is much higher than the most of vegetables and equivalent to mushrooms such as Shiitake and Enoki [30]. The presence of ergothioneine, a sulphur-containing amino acid, might bear relevance to the habitat of *Galdieria*, which is sulphur springs.

A polysaccharide extracted from *Galdieria* was also suggested to improve intestinal bacteria diversity and to increase the proportions of “beneficial bacteria” such as *Bifidobacterium*, *Coprococcus* and *Blautia*. Improved bacteria diversity has been demonstrated to be associated with remedial effects against various diseases such as inflammatory bowel disorder, depression, Alzheimer’s disease and cancer [31,32,33]. *Bifidobacterium*, *Coprococcus* and *Blautia* are known to improve the immune system, alleviate inflammation and increase muscle mass [34,35]. *Galdieria*-derived polysaccharide potentially functions as a prebiotic; i.e., compounds that promote the growth or activity of microorganisms in the large intestine, promoting good health. Our in-house results require verification by clinical trials.

*Galdieria* is also known to contain other beneficial metabolites such as a strong antioxidants, glutathione and a natural blue colourant, phycocyanin [14,15,26,36]. It is well-known that the levels of metabolites change depending on trophic modes; major metabolic shifts of metabolites such as pigments and lipids are reported in autotrophic cultivation [37,38,39,40,41,42]. Tuning the cultivation mode to one suitable for the metabolites of interest and the extraction of these metabolites could further contribute to the increased business potential of *Galdieria* as well as a reduction in the carbon footprint.

## 4. Materials and Methods

### 4.1. Galdieria Cultivation and Adsorbent Preparation

*Galdieria* sp. NS3 was sampled in Japan and isolated by Seed Bank Co., Ltd. (Kyoto, Japan). In heterotrophic and mixotrophic cultivation modes, *Galdieria* cells were grown at 42 °C with constant aeration in modified Allen’s media containing (in 1 L) 540 mg KH_2_PO_4_, 100 mg MgSO_4_·7H_2_O, 12 mg FeSO_4_·7H_2_O and 5.24 g (NH_4_)_2_SO_4_ with 4 mL/L of a concentrated modified A2 trace element solution containing (in 1 L) 1.8 g MnCl_2_·4H_2_O, 315 mg ZnCl_2_, 1.17 g Na_2_MoO_4_·2H_2_O, 40 mg CoCl_2_·6H_2_O and 55 mg CuCl_2_. 38.5 g caster sugar was supplemented as a carbon source and the pH was adjusted to 2.5 with concentrated H_2_SO_4_. In autotrophic cultivation, modified Allen’s media containing (in 1 L) 540 mg KH_2_PO_4_, 500 mg MgSO_4_·7H_2_O, 8 mg FeSO_4_·7H_2_O and 2.62 g (NH_4_)_2_SO_4_ with 2 mL/L of a concentrated modified A2 trace element solution containing (in 1 L) 5.4 g MnCl_2_·4H_2_O, 315 mg ZnCl_2_, 1.17 g Na_2_MoO_4_·2H_2_O, 120 mg CoCl_2_·6H_2_O and 129 mg CuCl_2_ was used with 2 L/minute CO_2_ bubbling. In mixotrophic and autotrophic cultivation modes, continuous LED light of 75–320 mmol photon/m^2^/s was used to illuminate the side of the cultivation tank, depending on the density of the cells. The cells were quantified using a spectrophotometer (AS ONE ASV11D-H, Osaka, Japan) as O.D._750_, harvested and dried in an oven at 50–70 °C (for metal adsorption) or in a lyophiliser (for content analysis). The dried, heterotrophically grown cells were ground to a fine powder and used as a metal biosorbent.

### 4.2. Metal Recovery and Quantification

Metal solutions were prepared by dissolving HAuCl_4_·3H_2_O, Pd(NO_3_)_2_·2H_2_O, Cu(NO_3_)_2_·3H_2_O, FeCl_3_·6H_2_O, NiCl_2_·6H_2_O, SnCl_2_·2H_2_O or ZnCl_2_. Aqua regia was prepared by mixing a three-to-one molar ratio of 35% (mass/mass) HCl and 61% (mass/mass) HNO_3_ and diluting with distilled water, the acidity of which was described as active aqua regia (non-diluted) to be 12 M. The indicated amount of the *Galdieria*-derived adsorbent was added to the indicated concentrations of Au or Pd solutions, vigorously vortexed to obtain a homogeneous suspension for 5 min, then centrifuged at 10,000 rpm at room temperature for 3 min unless otherwise stated. The activated charcoal and ion exchange resin used were JUNSEI first grade (JUNSEI, Tokyo, Japan) and TULSION A-23 (anion type, Thermax, Pune, India), respectively. An iodine solution was prepared by adding 3 g/L I_2_ and 6 g/L KI to distilled water. Fine-ground ores from Kenya were incubated overnight in 20 volumes of the iodine solution on a rotator to extract Au.

The solutions before and after adsorption were quantified for metal concentrations using an inductively coupled plasma optical emission spectrometer (ICP-OES) (Agilent5800 VDV; Agilent Technologies, Tokyo, Japan).

### 4.3. Content Analysis

Lyophilised cells grown in heterotrophic, mixotrophic and autotrophic cultivation modes were ground with a mortar and pestle. Amino acids, including ergothioneine, were analysed at Japan Food Research Laboratories (https://www.jfrl.or.jp/, accessed on 2 June 2023) using an amino acid automatic analyser and HPLC (for tryptophan and ergothioneine). The rest were analysed at the Laboratory of Food Environment and Hygiene (https://www.shokukanken.com/, accessed on 10 February 2023) using the loss-on-drying method (moisture), modified macro-Kjeldahl method and Dumas method (protein), acid digestion (fat), Prosky method (fibre), direct ashing method (ash), ICP-OES (sodium) and modified Atwater method (energy).

### 4.4. qRT-PCR Analysis

Total RNA was extracted from *Galdieria* sp. NS3 grown in heterotrophic, mixotrophic and autotrophic modes using a phenol:chloroform:isoamyl alcohol (Sigma-Aldrich, Burlington, MA, USA) method, as described elsewhere [43]. RNA was purified using sodium acetate (Sigma-Aldrich), precipitated and treated with DNase I (Invitrogen, Waltham, MA, USA), followed by cDNA synthesis using SuperScript III (Invitrogen) according to the manufacturer’s instructions.

qRT-PCR was performed using the SYBR Green (THUNDERBIRD, TOYOBO, Osaka, Japan) method and an AriaMx real-time PCR system (Agilent). The PCR cycle was 95 °C for 1 min prior to 40 cycles of 95 °C for 15 s and 60 °C for 30 s, followed by dissociation steps for a melting curve. *Actin* (Gasu_15600) was employed as a housekeeping gene. The primers used were *argininosuccinate synthase* forward ATGGTTGGGTGGATGTATTGTGTTAC, reverse CCATACTTGCCAACTTATCGCTGTAC and *actin* forward GCTCCATTTTGGCGAGTCTCAG, reverse CATCATATTCCTCTCTTGTGACCCAC.

### 4.5. Statistical Analysis

Statistical differences were determined using one-way ANOVA tests with Sidak’s multiple comparison post-tests or a *t*-test with Welch’s correction using Prism (GraphPad 10.1.2 Software).

## 5. Conclusions

The red microalga *Galdieria* has great potential for commercialisation as (1) it can grow quickly to a high cell density with a low risk of biological contamination; (2) it has precious metal adsorption ability; and (3) it contains various high-value metabolites. Many start-up companies have abandoned the algae industry because the mass production of algae on a commercial scale to generate profits without biological contamination is simply too challenging. As *Galdieria* is an extremophile that thrives in strong acidity and moderately high temperatures, the risk of other organisms contaminating the *Galdieria* growth medium is low. Additionally, *Galdieria* can grow in autotrophic, mixotrophic and heterotrophic modes. In a heterotrophic mode, it can utilise various carbon sources and can grow to a high cell density, producing quantities sufficient for commercialisation. Our *Galdieria*-derived metal adsorbent is suitable for the recovery of precious metals such as Au and Pd as a natural property of the cell surface of *Galdieria* and the adsorbent does not need extensive pretreatments or processing. *Galdieria* selectively and efficiently collects precious metals from base metals, especially in dilute solutions, and performs well in extremely strong acids such as 6 M aqua regia. As this precious metal adsorption event occurs on the surface of the cell, the cell content of *Galdieria* can be utilised for other purposes such as nutrients, cosmetics and pharmaceuticals. *Galdieria* has been shown to be protein-rich and contains beneficial substances such as ergothioneine and glycogen. There is also a possibility that target metabolites can be preferentially produced by shifting the modes of growth. By removing these carbon-rich metabolites, the adsorption capacity of the adsorbent per weight increases and carbon emissions are reduced through combustion at the end of the metal collection process. Altogether, *Galdieria* is a promising and sustainable alternative compared with currently used metal adsorbents or methodologies as it is efficient for Au and Pd recovery, especially in low metal concentrations; highly tolerant to acidity; and selective towards precious metals among base metals. By recovering metals from dilute wastewater that is discarded at present using the *Galdieria* adsorbent, the metal recycle ratios could be improved further, contributing to a circular economy.

## Figures and Tables

**Table 1:** Cell component composition of *Galdieria* grown in each trophic mode.

	Heterotrophy	Mixotrophy	Autotrophy	Unit
Moisture	4.2	2.3	1.5	g
Protein	37.9	32.0	64.2	g
Fat	4.9	4.2	8.3	g
Carbohydrate	50.6	59.8	22.8	g
- Fibre	39.2	42.1	22.8	g
- Sugar	11.4	17.7	0.0	g
Ash	2.4	1.7	3.2	g
Sodium	0.028	0.012	15	mg
- SCE ^1^	0.071	0.030	0.038	g
Energy	320	321	377	kcal

All values in per 100 g dry weight. ^1^ Sodium chloride equivalent.

**Table 2:** Amino acid profile of *Galdieria* grown in each trophic mode.

	Heterotrophy	Mixotrophy	Autotrophy
Asp + Asn	2.16	2.36	5.49
Ala	1.41	1.56	3.99
Arg	1.37	1.66	3.75
Ile	1.24	1.37	3.15
Gly	1.17	1.22	2.60
Glu + Gln	4.34	4.64	8.71
Cys	0.74	0.74	1.28
Thr	1.90	2.01	3.56
Ser	2.17	2.21	4.16
Tyr	1.85	2.03	3.92
Trp	0.40	0.41	0.73
Val	1.79	1.87	3.66
His	0.51	0.53	0.77
Phe	1.15	1.22	2.47
Pro	1.49	1.56	2.61
Met	0.52	0.58	1.45
Lys	1.91	2.04	3.54
Leu	1.85	2.04	4.65
**Sum**	**27.97**	**30.05**	**60.49**

All values in g/100 g dry weight.

**Table 3:** Ergothioneine concentrations in *Galdieria* grown in each trophic mode.

Heterotrophy	Mixotrophy	Autotrophy
36	35	39

All values in mg/100 g dry weight.

## Data Availability

The datasets generated and/or analysed in this study are available from the corresponding author upon reasonable request.
